# Response of Psychrotolerant Fungus *Mucor flavus* to Cell Wall Stress, Induced by Azo Dyes

**DOI:** 10.3390/ijms27114927

**Published:** 2026-05-29

**Authors:** Sofiya A. Saharova, Elena A. Ianutsevich, Olga A. Danilova, Galina A. Kochkina, Vera M. Tereshina

**Affiliations:** 1 Winogradsky Institute of Microbiology, Research Center of Biotechnology of the Russian Academy of Sciences, 33, Bld. 2 Leninsky Ave., 119071 Moscow, Russia; saharovasofia814@gmail.com (S.A.S.); noitcelfer@mail.ru (O.A.D.); v.m.tereshina@inbox.ru (V.M.T.); 2 Skryabin Institute of Biochemistry and Physiology of Microorganisms, Federal Research Center “Pushchino Centre for Biological Research,” Russian Academy of Sciences, 5, pr. Nauki, 142290 Pushchino, Russia; gaga56@mail.ru

**Keywords:** cell wall stress, Congo red, calcofluor white, phosphatidic acids, chitin, chitosan

## Abstract

The cell wall (CW) of *Mucoromycota* has a unique chitin/chitosan complex, unlike chitin/glucan complex in *Ascomycota*. Under cell wall stress (CWS), induced by azo dyes, ascomycetes increase the amount of CW chitin. This study analyzes the response of *Mucor flavus* to CWS, induced by Congo red and Calcofluor white. It was found that azo dyes significantly reduced the biomass yield and inhibited apical growth and branching but did not lead to an increase in the amount of CW chitin/chitosan, neutral polysacchrides and cytosol osmolytes. Non-bilayer phosphatidic acids and phosphatidylethanolamines dominated in the control membrane lipids, but the proportion of bilayer phosphatidylcholines did not exceed 5%. Under CWS, the proportion of phosphatidic acids increased, while the proportion of phosphatidylethanolamines decreased and the degree of unsaturation of phospholipids increased. Storage lipids in the control were represented by mono-, di- and triacylglycerides and free fatty acids. Under CWS, the proportion of diacylglycerides increased significantly, while the proportion of triacylglycerides decreased. Thus, the CWS response of *M. flavus* consisted of significant changes in growth and the composition of membrane and storage lipids, but the amount of CW chitin/chitosan and cytosol osmolytes did not increase, which is different from the response of ascomycetes.

## 1. Introduction

The fungal cell wall (CW) is a multifunctional structure that maintains cell shape and protects against mechanical and osmotic stress. It is also responsible for biotic and abiotic interactions [1,2]. The composition and structure of the CW change both during the life cycle of fungi and in response to stress, starvation, and mutualistic and parasitic interactions.

The main components of the fungal CW are polysaccharides, including various glucans (with β-1,3-, β-1,3-/β-1,4-, β-1,6-, and α-1,3-linkages), chitin and chitosan, mannan and/or galactomannan, and glycoproteins [3]. The CW of ascomycetous and basidiomycetous fungi is rich in glucans (mainly β-1,3-glucan) and chitin, while chitosan is absent. In the pathogenic fungus *Aspergillus fumigatus*, linear polymers, chitin, and partially α-1,3-glucan form microfibrils [4], which are covalently linked to other polysaccharides (galactomannan, galactosaminogalactan, and β-1,3-1,4-glucan) [5]. The rigid chitin microfibrils of *A. fumigatus* do not form a continuous layer and are localized in the inner layer of the CW, where they are covalently linked to a flexible branched β-1,3-glucan with β-1,6-side branches [6].

Compared to *Ascomycota* and *Basidiomycota* the composition and structure of the CW of *Mucoromycota* are poorly studied. The main components of the CW of mucoromycetous fungi are chitin (a linear homopolymer of N-acetyl-D-glucosamine with β-1,4-linkages) and chitosan (a linear homopolymer of D-glucosamine with β-1,4-linkages). However, the presence of chitosan as a separate component rather than a partially deacetylated form of chitin is a controversial issue. It has been previously suggested that the deacetylation of chitin in the CW is carried out by a chitin deacetylase unique to *Mucoromycota* [7], but later chitin deacetylase genes were discovered in many ascomycetes and basidiomycetes [8]. The CW of the plant-pathogenic hemibasidiomycete *Ustilago maydis* lacks polyuronides and chitosan [9]. However, this fungus has three chitin deacetylase genes, and the mutation of one of them has led to a loss of virulence and the inability to transform into a mycelial form, indicating that chitosan plays an important role in the life cycle of this phytopathogen [8].

The second significant component of the CW is the heteropolysaccharide glycuronan, which consists of fucose, galactose, mannose, and glucuronic acid in a ratio of 5:1:1:6 [10]. Glycuronan consists of two components: the difficult-to-hydrolyze mucoric acid (a polymer of glucuronic acid) and the easily hydrolyzable mucoran, which consists of glucuronic acid, fucose, and mannose in a ratio of 5:2:3. It is believed that β-glucan is unique to the CW of the spores but not hyphae of mucoromycetes [7,11,12,13]. Microorganisms require robust defense mechanisms to survive a variety of stressors. Stress is the state of the microorganisms under the influence of biotic or abiotic factors, which trigger the defense system. The stress response is divided into acute and adaptive phases. Acute phase aims to minimize damage, while adaptive phase aims to restore homeostasis to ensure growth under new conditions [14]. In the life cycle of fungi, cell wall stress (CWS) is caused by the action of sexual pheromones [1], resulting in sexual morphogenesis. In addition, CWS occurs as a result of certain stressors and chemical compounds that disrupt the synthesis or assembly of the main polysaccharides of the CW. These compounds include fungicidal antibiotics, azo dyes, lytic enzymes, and antibiotics that inhibit β-1,3-glucan and chitin synthases [15]. The response to CWS has not been sufficiently systematically studied; the main marker is considered to be an increase in chitin level in the CW, associated with the activation of GlcN-6-P synthase *(gfaA)*, which catalyzes the first rate-limiting stage of chitin synthesis—the formation of D-glucosamine-6-P from D-fructoso-6-P and L-glutamine [15,16]. But some researchers believe that the *agsA* gene, encoding α-glucan synthase, may be a marker of the response to CWS in aspergilli. However, in mucoromycetes, there are no glucans in the hyphal CW and they may have a different marker [17]. Unfortunately, unlike aspergilli, there are no studies of the transcriptome of mucoromycetes under CWS conditions.

Azo dyes Congo red (CR) and calcofluor white (CFW) are used to create CWS in vitro. It is believed that CFW binds more strongly to chitin, while CR binds more strongly to β-1-3- and α-1-3-glucans [18,19]. It is known that CR binds well with cellulose and carboxymethylcellulose, which is used to determine the activity of cellulolytic enzymes in microorganisms [20].

It is believed that the sublethal concentrations of antifungals, which induce a morphological response in *A. niger* germlings, including the formation of (sub)apical branches, reflect the fungus’s adaptation to CWS [17,21]. The action of azo dyes leads to morphological changes, especially in the early stages of growth, where active synthesis of the CW occurs. For instance, incomplete separation of mother and daughter cells is observed in yeast, while swelling and lysis of the apical ends of hyphae occur in mycelial fungi [15]. Alternatively, the apical growth may stop, resulting in multiple hyphal extensions in *A. niger* [22]. However, it has been established that the action of CR affects not only the synthesis of the CW but also affects primary and especially secondary metabolism, demonstrating its pleiotropic effect [19].

In response to CWS, the cell wall integrity (CWI) pathway is activated in fungal cells, leading to increased synthesis of the main polysaccharides of the CW, such as chitin and glucan [22,23]. The CWI pathway plays a crucial role in morphogenesis, virulence, and antifungal susceptibility [24]. The CWI pathway is mediated by the MtlA sensor protein [25] and a multi-step phosphorylation system involving the induction of RhoB and RhoD GTPases through the mitogen-activated protein kinase-kinase MAP2K and the transcription factor RlmA [21].

In yeast and aspergilli, two additional pathways respond to general stress and CWS, in addition to the CWI pathway. In yeast, the response is mediated by the transcription factors Msn2p/Msn4p and, in aspergilli, by the ortholog of MsnA, as well as by the calcium/calcineurin pathway, mediated by the factors Crz1p and CrzA, respectively [26]. In addition, it has been shown that the MpkC and SakA proteins from *A. fumigatus*, which are homologs of the Hog1 protein from *Saccharomyces cerevisiae*, are involved in adaptation to oxidative and osmotic stress, heat shock, and CWS [27]. SakA and MpkC interact with 118 and 213 proteins under osmotic stress and 420 and 299 proteins under Congo red induced CWS, respectively. The co-immunoprecipitation method has been used to demonstrate the interaction of MpkC and SakA with multiple proteins involved in signal transduction, CW synthesis (UDP-N-acetylglucosamine diphosphorylase, N-acetylphosphoglucosamine mutase, and 1.3-beta-glucanosyltransferase), lipid (ergosterol synthesis enzymes) and carbohydrate metabolism (trehalase phosphorylase and trehalose-6-phosphate synthase), as well as protein folding (HSP 30, HSP 70, and HSP 90). Additionally, SakA::GFP is physically associated with MpkC:3xHA under osmotic stress and CWS, as well as with cell wall integrity MAPK MpkA:3xHA, according to co-immunoprecipitation assays.

These pathways are also involved in adapting to many abiotic stressors, including changes in the composition of cytosolic osmolytes and membrane lipids. However, a study of the composition of osmolytes and membrane lipids in *Aspergillus niger* showed that CWS, induced by azo-dyes, caused significant morphological changes in the hyphae of the fungus and inhibition of apical growth and an increase in the amount of chitin and glucan in the CW, while the composition of membrane lipids did not change significantly, but the degree of their unsaturation increased markedly. Additionally, the changes in the composition of osmolytes were not adaptive in nature [22].

Previously, *Mucor flavus* was classified as a psychrotolerant due to its wide growth range of −2 to 27 °C, with an optimum of 20–22 °C [28]. This fungus is an attractive object for studying the effect of azo dyes that cause CWS due to its high growth rate, extremophilia, and affiliation to *Mucoromycota*. Unlike *Ascomycota*, fungi of *Mucoromycota* do not have glucans in the CW of the hyphae.

The objective of this study is to examine the effect of CWS induced by azo dyes CR and CFW on the growth, composition of osmolytes, membrane lipids, and content of the main CW polysaccharides in the psychrotolerant fungus *M. flavus*.

## 2. Results

### 2.1. The Effect of Azo Dyes on M. flavus Mycelium Morphology

**Figure 1:**
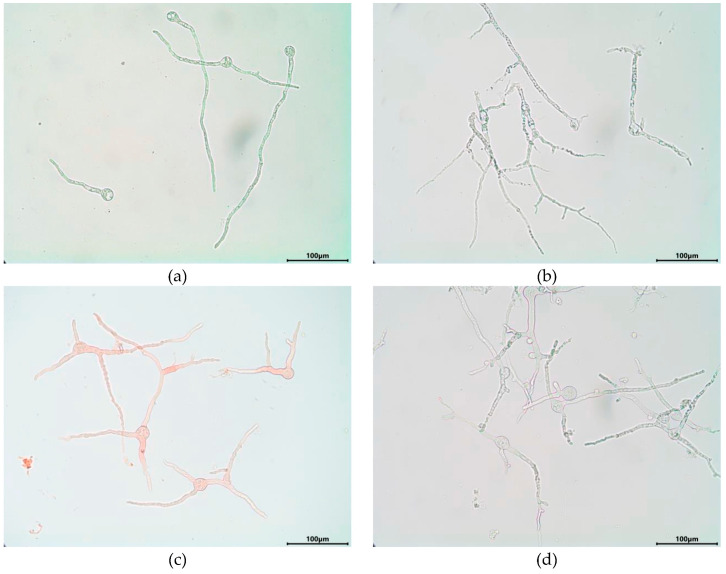
*M. flavus* growth in submerged culture under optimal conditions after 12 h (**a**) and 16 h (**b**) of cultivation and 4 h after adding 100 μg/mL of Congo Red (CR) (**c**) or 75 μg/mL of calcofluor white (CFW) (**d**). Scale bar is 100 μm.

To develop the experimental protocol, concentrations of azo dyes ranging from 25 to 150 μg/mL were used. As a result, the concentrations that arrest apical growth of the fungus were selected, being 100 μg/mL for CR and 75 μg/mL for CFW. Cultivation was carried out using Goodwin’s medium with a pH of 6.0. It was shown that the pH remained stable during the experiment, which is crucial for maintaining the soluble form of azo dyes (at a pH > 5.5) [18]. The spores germinated with 1–4 germ tubes. In the control, all spores germinated by 12 h, forming long, non-branching germ tubes (Figure 1a). After 4 h, active branching of the growth tubes was observed (Figure 1b). CR and CFW significantly inhibited biomass accumulation and prevented germ tubes branching, without swelling of the growth tubes or lysis at their apical ends (Figure 1c,d). CR provides a reddish hue to the CW and cytosol (Figure 1c). In the control, 1.03 ± 0.05 g/L of biomass had accumulated by the end of the experiment. The effects of CR (0.58 ± 0.03 g/L) and CFW (0.41 ± 0.02 g/L) significantly slowed the growth of the fungus.

### 2.2. The Effect of Azo Dyes on the Composition of the Main Polysaccharides of the M. flavus Cell Wall

**Figure 2:**
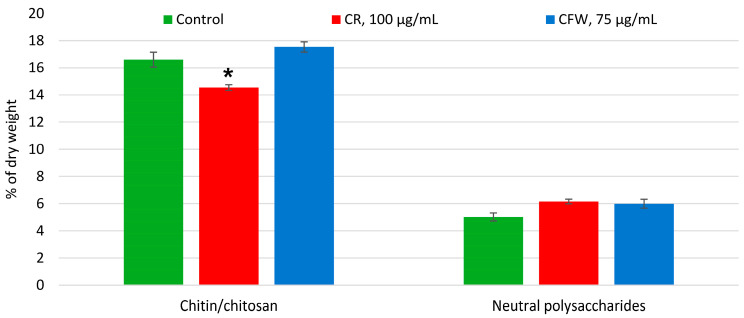
Chitin/chitosan and neutral polysaccharides content of *M. flavus* cell wall (% of the cell wall dry weight) in the presence of CR and CFW. Means ± SEM are displayed, *n* = 3, SEM—standard error of the mean. Statistically significant difference (*p* ≤ 0.05) is indicated by (*).

The CW of *M. flavus* contained the aminopolysaccharides chitin/chitosan (16% of the CW) and neutral polysaccharides (5% of the CW) (Figure 2). Under the influence of CFW (blue column), the amount of chitin/chitosan did not change significantly compared to the control. In contrast, the effect of CR (red column) led to a 12% decrease in the amount of chitin/chitosan. Meanwhile, both azo dyes did not significantly change the amount of neutral polysaccharides.

### 2.3. The Effect of Azo Dyes on the Composition of M. flavus Osmolytes

**Figure 3:**
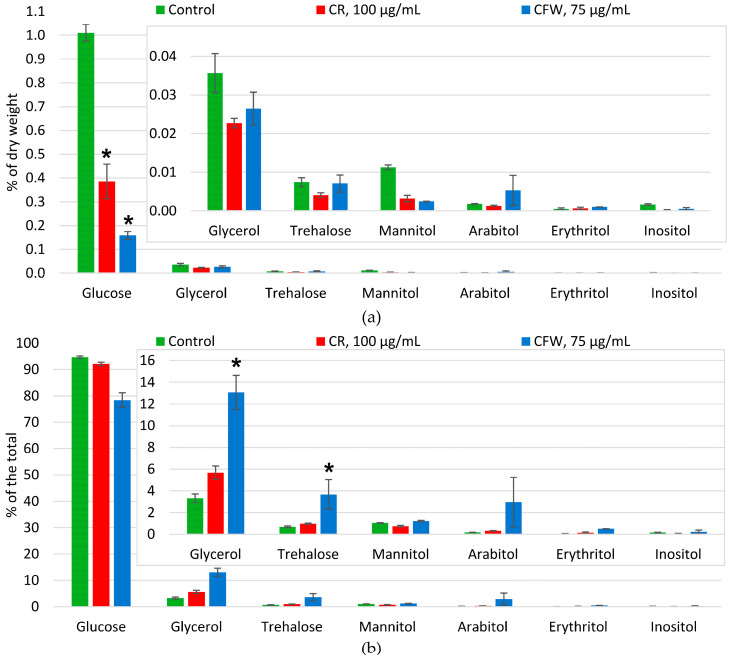
The effects of cell wall stress (CWS), induced by CR and CFW, on the composition of soluble carbohydrates and polyols of *M. flavus*. (**a**)—% of dry weight; (**b**)—% of the total. Means ± SEM are displayed, *n* = 3, SEM—standard error of the mean. Statistically significant difference (*p* ≤ 0.05) is indicated by (*).

In the control variant, glucose was the dominant soluble carbohydrate in the cytosol (1% of the dry weight and 95% of the total) (Figure 3a,b). Glycerol, erythritol, arabitol, mannitol, inositol, and trehalose were present in trace amounts (less than 5% of the total). The amount of soluble carbohydrates and polyols in the control variant was approximately 1.1% of the dry weight. This amount decreased by a factor of three under the influence of CR and by a factor of five in the presence of CFW due to a decrease in glucose level. The ratio of components changed slightly under the influence of CR, while CFW decreased the proportion of glucose to 78%, increased the proportion of glycerol to 12%, and increased the proportion of trehalose to 4% (Figure 3b). This resulted in a significant decrease in the glucose/glycerol ratio in the CFW variant.

### 2.4. The Effect of Azo Dyes on the Composition of M. flavus Storage and Membrane Lipids and Their Fatty Acids

**Figure 4:**
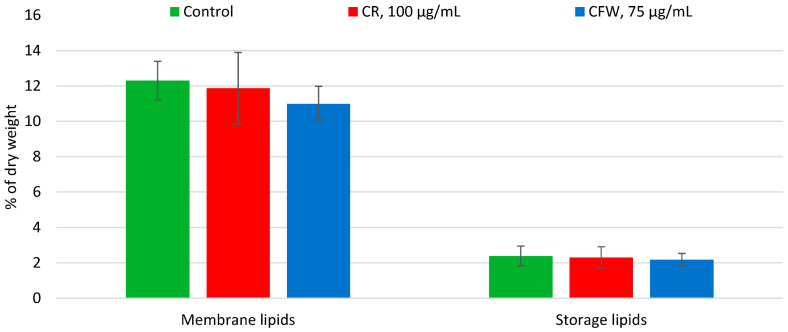
Effect of the CWS on the levels of membrane and storage lipids of *M. flavus*. Means ± SEM are displayed, *n* = 3, SEM—standard error of the mean.

**Figure 5:**
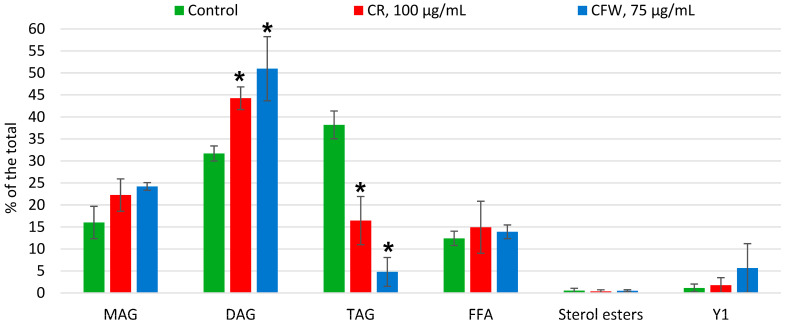
Effect of the CWS on the composition of *M. flavus* storage lipids. Means ± SEM are displayed, *n* = 3, SEM—standard error of the mean. Statistically significant difference (*p* ≤ 0.05) is indicated by (*). MAG—monoacylglycerols, DAG—diacylglycerols, TAG—triacylglycerols, FFA—free fatty acids, Y1—unidentified lipid.

Both azo dyes did not change the amount of storage lipids (2.5% of dry weight) in comparison with control (Figure 4). The main storage lipids in the control were di- and triacylglycerols (DAG and TAG, respectively), reaching 70% of the total storage lipids (Figure 5). A marked increase in the DAG proportion is observed under CWS, induced by CR and CFW, accompanied by a decrease in the TAG proportion.

**Figure 6:**
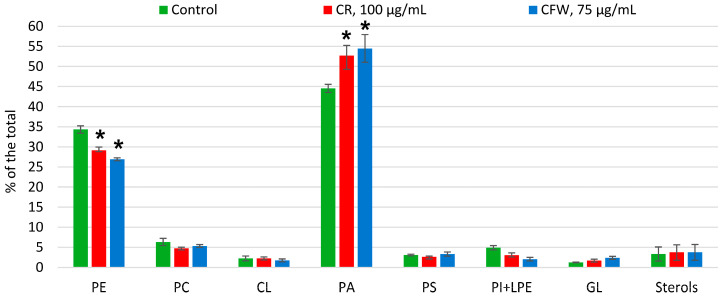
Effect of the CWS on the composition of *M. flavus* membrane lipids. Means ± SEM are displayed, *n* = 3, SEM—standard error of the mean. Statistically significant difference (*p* ≤ 0.05) is indicated by (*). PE—phosphatidylethanolamines, PC—phosphatidylcholines, CL—cardiolipins, PA—phosphatidic acids, PS—phosphatidylserines, PI—phosphatidylinositols, LPE—lysophosphatidylethanolamines, GL—glycolipids, St—sterols.

The amount of membrane lipids in the control group reached 12% of the dry weight and did not change in response to the influence of both azo dyes (Figure 4). The membrane lipids of the fungus in the control variant were dominated by phosphatidic acids (PA) (44% of the total) and phosphatidylethanolamines (PE) (34% of the total), while the proportions of phosphatidylcholines (PC) and sterols did not exceed 5% (Figure 6). The remaining phospholipids (cardiolipins, phosphatidylserines, phosphatidylinositols, and lysophosphatidylethanolamines), as well as glycolipids, were present in trace amounts. Under the influence of both azo dyes, the proportion of PA increased to 54%, while the proportion of PE decreased.

**Figure 7:**
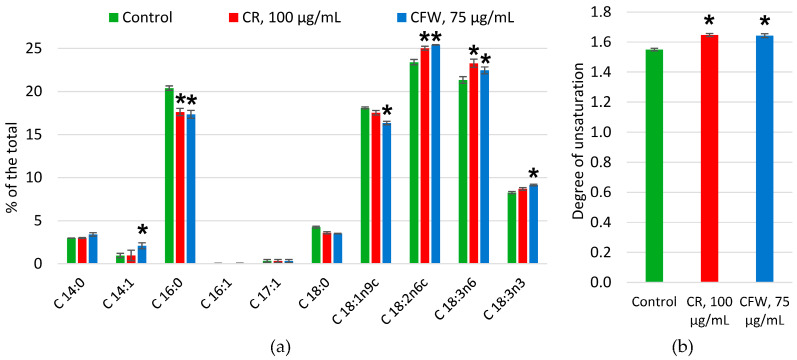
Effect of the CR and CFW on the composition of the main fatty acids (**a**) and degree of unsaturation (**b**) of *M. flavus* membrane phospholipids. Means ± SEM are displayed, *n* = 3, SEM—standard error of the mean. Statistically significant difference (*p* ≤ 0.05) is indicated by (*).

The fatty acids of the membrane phospholipids were represented by approximately equal parts (18–25% of the total) of palmitic (C16:0), oleic (C18:1), linoleic (C18:2), and γ-linolenic (C18:3) acids (Figure 7a). The presence of both azo dyes resulted in a modest increase in the proportions of linoleic and linolenic acids, accompanied by a decrease in the proportion of palmitic acid. This led to a marginal increase in the degree of unsaturation (DU), as measured by the increase from 1.55 in the control to 1.65 in the CR and CFW groups (Figure 7b).

## 3. Discussion

Goodwin’s medium with a pH of 6.0 was used for the cultivation of the fungus. It was demonstrated that the pH remained constant throughout the duration of the experiment. This is important because CR and CFW, which have two sulfonyl groups, become insoluble at a pH below 5.5 [18,29]. The azo dyes were introduced at a growth point of 12 h, when all the spores had germinated and formed long germ tubes but had not yet begun to branch out. In the control, active branching of the germ tubes was observed after 4 h. Under the influence of both azo dyes, growth and branching processes were inhibited; however, no swelling was observed along the length of the germ tubes, nor was any swelling of the apices or their lysis observed. In comparison with the control, the amount of biomass was reduced by half in the azo dyes groups. The data presented herein illustrate the differential impact of azo dyes on the germination process of mucoromycetous and ascomycetous fungi. In the ascomycete *A. niger*, the azo dyes caused the apical growth to stop, similar to the mucoraceous fungus. However, the morphology of the germ tubes was significantly altered, with the appearance of numerous globular swellings [22].

The CW structure of *Mucoromycota* is poorly studied, and only limited data have been obtained for pathogenic fungi of the genera *Mucor* and *Rhizopus* [30,31]. Using multidimensional solid-state NMR analysis, it was demonstrated that hyphal CW contain a highly polymorphic chitin/chitosan complex that forms a rigid layer, as well as matrix components made up of protein, lipids, and neutral polysaccharides based on fucose, mannose, and galactose [30]. However, glucan content is very low, and chitosan is present not as a separate polymer but as regions of partially deacetylated chitin. The authors believe this contributes to the preservation of the CW hydration and dynamics. Notably, this work does not mention glucuronic acid polymers, as reported earlier [10]. A study of the immunomodulatory potential of spores and germ tubes of the pathogenic fungi *Rhizopus arrhizus*, *M. circinelloides*, and *Lichtheimia corymbifera* using staining and lectin- and immunolabeling techniques yielded conflicting results. Spores and germ tubes were found to contain surface-localized mannan, and β-1,3-glucan was detected on the surface of germ tubes [31]. However, despite the fact that β-1,3-glucan is a branched polymer with β-1,6 side branches, the authors believe that glucose is the main sugar and β-1,3-glucan is a microfibrillar polymer [6]. It has been demonstrated that the main monosaccharides in the CW are glucose, mannose, galactose, and xylose [31], although only fucose, mannose, and galactose were previously found in *M. rouxii* as part of mucoran and mucoric acid [10]. It has also been proven by a radioactive method that fucose is incorporated into these polymers by fucosyltransferase [32].

Based on the above, it can be concluded that the CW of mucoromycetes is unique in that its main component is the chitin/chitosan complex. However, the full composition and structure of the CW remain unknown. Our work yielded unexpected results compared to previous studies on *A. niger*. In those studies, the effects of CR and CFW were shown to significantly inhibit apical growth, cause expansions in hyphae, and increase the amount of chitin and glucan in the CW [22]. These results are consistent with molecular biology studies that show that *A. niger* responds to CWS by activating the *agsA* and *gfaA* genes via a transcription factor similar to Rlm1p [33]. In contrast, neither azo dye affected the hyphae of *M. flavus*, although both significantly reduced biomass yield and inhibited apical growth and branching. Neither dye increased the amount of chitin/chitosan in the CW. Furthermore, CR had the opposite effect, decreasing its level. It should be noted that both azo dyes did not affect the neutral polysaccharide content. A unique feature of the CW of mucoraceous fungi is the absence of flexible glucans and the presence of chitin and chitosan. This suggests that they may have a different mechanism for responding to CWS, such as a change in the ratio of these aminopolysaccharides, or a change in the amount and composition of another CW component, glycuronan. Other researchers have also observed the resistance of mucoromycetes to azo dyes [34]. The lack of glucan in the CW explains the resistance to CR, which binds more strongly to glucans. The authors propose that resistance to CFW suggests the presence of other mechanisms unrelated to chitin synthesis. Alternatively, the CW resistance to azo dyes may be related to the fungus’ extremophilic nature.

Osmolytes, which in fungi mainly include the disaccharide trehalose and polyols, are known to be involved in adaptation to many abiotic stressors [35,36,37] and play a significant role in fungal thermophilia [38], alkalophilia [39], acidophilia [40,41], xerophilia [42], and psychrophilia [43,44]. However, analysis of the composition of soluble carbohydrates and polyols in the cytosol of *M. flavus* revealed that osmolytes are not involved in adaptation to CWS caused by CR and CFW. Their amount was very low (approximately 1.1% of dry weight), decreased several times due to a drop in glucose levels when azo dyes were present, and, most importantly, trehalose and polyols did not accumulate in noticeable amounts in the composition of carbohydrates and polyols in the cytosol (Figure 3). Only one change in the ratio of carbohydrates and polyols in the cytosol was observed under the influence of CFW, specifically, a noticeable increase in the proportion of glycerol and trehalose. However, the low glycerol amount does not allow us to discuss its protective effect. Previously, we demonstrated that, after 24 h of cultivation, *M. flavus* accumulates approximately 2% of soluble carbohydrates in its mycelium, consisting of glucose and trehalose at a 2:1 ratio [28]. After 48 h, the carbohydrate content increased to 2.5%, with a ratio of 1:3; thus, trehalose emerged as the primary protective compound of this psychrotolerant fungus. Trehalose is a multifunctional compound with protective, antioxidant, transport, reserve, and chaperone functions [45,46,47,48,49,50]. The primary function of trehalose in cells is considered to be protective. Unlike polyols, this disaccharide protects both the cytosol macromolecules and the membranes. However, we did not observe a significant increase in trehalose levels in the studied fungus under the influence of azo dyes (Figure 3). Conversely, glucose levels decreased significantly, consistent with the inhibition of growth processes.

Branching of the germ tubes indicates the end of germination and the formation of young mycelium. At this growth stage, *M. flavus* is characterized by a high concentration of membrane lipids (11–12% of dry biomass), regardless of the azo dye effect. Psychrotolerant fungus is characterized by a low proportion of sterols and glycolipids, as well as a high proportion of phospholipids (95% of the total). In the control, the phospholipids were dominated by non-bilayer PA (45% of the total) and PE (35%), while the proportion of bilayer PC did not exceed 5% (Figure 6). A similar pattern was observed in *A. niger* at the same growth stage [22]. However, the azo dyes did not affect the composition of *A. niger* membrane lipids, while, in *M. flavus*, the azo dyes increased the proportion of PA and decreased the proportion of PE (Figure 6). Furthermore, the azo dyes did not affect the composition of the storage lipids in *A. niger*, which were dominated by FFA (55–65% of the total). In the control sample of *M. flavus*, however, the storage lipids were composed of TAG (37% of the total), DAG (32% of the total), MAG (15% of the total), and FFA (15% of the total). The azo dyes significantly decreased the proportion of TAG and increased the proportion of DAG. These data suggest that the azo dyes noticeably affect the composition of both membrane and storage lipids in *M. flavus* but not in *A. niger*.

A common pattern in the early trophophase stage of *A. niger* [22] and *M. flavus* is the predominance of PA in membrane lipid composition and the predominance of non-bilayer phospholipids. The proportion of PA and PE in *A. niger* reaches 70%, while, in *M. flavus*, it reaches 78%. PA are multifunctional compounds that are key intermediates in the synthesis and metabolism of phospholipids and reserve lipids [51]. Due to their conical shape and tendency to aggregate, PA microdomains can form negative curvature of membrane. This determines their role in membrane structure, fusion, division, and the processes of membrane traffic, endocytosis, and exocytosis [52,53,54]. PA’s negative charge stabilizes protein–lipid interactions when it interacts with lysine or arginine. There are four distinct mechanisms for membrane fusion and fission. PA is a substrate for enzymes producing DAG and lysophosphatidic acid; PA microdomains generate membrane curvature; PA interacts with proteins involved in fission or fusion; PA activates enzymes required for membrane rearrangements [55].

A high level of PA has been observed in membrane lipids in response to abiotic stressors and in extremophiles [39,40,42,43,56,57]. The term “lipid morphogen” has been proposed for such non-bilayer lipids that play an important role in the dynamic organization of membranes under stress [58]. In contrast to *A. niger*, where the composition of membrane lipids remained unchanged, the observed increase in PA proportion in the membrane lipid composition of the studied fungus under azo dye influence suggests its involvement in CWS adaptation.

It has previously been shown that the composition of membrane lipids does not change in *A. niger* under the influence of azo dyes. However, the DU increases significantly due to an increase in the proportion of α-linolenic acid (C18:3) [22]. In *M. flavus*, we also observed an increase in the DU of phospholipids in the presence of CR and CFW; however, it was only 7% higher, than that of the control (Figure 7b). This does not allow us to confidently conclude that this mechanism is involved in adaptation to CWS. Additionally, azo dyes did not alter the sterol-to-phospholipid ratio. Combined with minor changes in fatty acid composition, this finding does not permit us to confidently propose an increase in membrane fluidity due to azo dye exposure [59,60].

The mechanism by which azo dyes act on fungal mycelium remains poorly understood. However, initial studies show that, even at a low concentration of 50 μg/mL, CR stimulates the expression of 432 genes and suppresses the expression of 271 genes in *A. fumigatus* [19]. Interestingly, CR did not affect the genes responsible for synthesizing chitin and β-1,3-glucan, but it did stimulate the synthesis of their precursors: N-acetyl-D-glucosamine and N-acetyl-D-galactosamine. In addition to affecting the composition of the CW, CR increased the expression of genes involved in primary metabolism; however, it had the most significant impact on the synthesis of secondary metabolites. Our studies on the effects of azo dyes on *A. niger* [22] and *M. flavus* did not reveal the involvement of the osmolytic system in adapting to the CW. Additionally, they did not detect an increase in chitin or chitosan in the CW. However, they did reveal various effects on lipid composition. In *A. niger*, the fatty acid composition of phospholipids changed under the influence of CR and CFW, while the composition of membrane and storage lipids remained constant. In contrast, *M. flavus* exhibited significant changes in membrane and storage lipid composition, as well as increased DU of membrane phospholipids.

Thus, the response of *M. flavus* to CWS, induced by CR and CFW, included growth inhibition and the halting of apical growth and branching. Although, it did not increase the amount of CW chitin/chitosan or cytosol osmolytes. However, CWS caused significant changes in the composition of membrane and storage lipids and increased the degree of unsaturation of membrane phospholipids.

## 4. Materials and Methods

### 4.1. Objects of Study and Cultivation Protocol

The mucoromycete *Mucor flavus* Bainier 1903 VKM F-4102D (All-Russian Collection of Microorganisms (VKM), Pushchino, Russia) (*Mucoraceae*, *Mucorales*, incertae sedis, *Mucoromycetes*, *Mucoromycota*, *Fungi*) was used. The fungus was isolated from the soil of the Ledyanaya Cave (Krasnoyarsk krai, Berezovsky district), where the air and soil temperatures were 4–6 °C. The LSU rDNA sequence of this strain was deposited in the GenBank with accession number OR575477.

The fungus was cultivated at the optimal temperature (20 °C) for 6–7 days on slanted malt agar (7° Balling) and stored at 4–8 °C.

Submerged cultivation of the fungus was carried out in 250 mL flasks with 50 mL of Goodwin medium [61] on New Brunswick Innova 44R shaker (Eppendorf, Enfield, CT, USA) at 150 rpm. The inoculum was a spore suspension added to a concentration of 10^6^–10^7^ spores/mL of the medium. Cultivation was performed at the optimal temperature of 20 °C for 12 h. Azo dyes were then added to the medium at final concentrations of 100 μg/mL for CR (Aldosa, Moscow, Russia) and 75 μg/mL for CFW (Himedia, Thane, MH, India), and cultivation continued for an additional 4 h. Control was maintained under the same conditions without the addition of azo dyes.

For biochemical analyses, the biomass was separated from the culture fluid using a nylon filter, then washed with distilled water. Excess moisture was removed, and the biomass was weighed and stored at −21 °C. Dry biomass was determined gravimetrically.

### 4.2. Lipids, Carbohydrates, and Polyols Analysis

Solvents and other reagents were of analytical grade and supplied by Dia-m, Rushim or Chimmed (all Moscow, Russia), unless stated otherwise. For the analysis of lipid composition, a weighed sample of wet biomass was homogenized in isopropanol and extracted for 30 min at 70 °C, and the supernatant was decanted. The precipitate was then extracted twice with a 1:1 mixture of isopropanol and chloroform, followed by one extraction with a 1:2 mixture of the same solvents under the same conditions (modified Nichols method) [62,63]. The combined extracts were dried using a rotary evaporator, and the residue was dissolved in 9 mL of a 1:1 chloroform–methanol mixture. To this, 12 mL of 2.5% NaCl solution was added to remove water-soluble compounds. After phase separation, the chloroform layer was collected, dried over anhydrous Na_2_SO_4_, and concentrated using a vacuum rotary evaporator. The residue was then dried to a constant mass under vacuum. Finally, the residue was dissolved in a 2:1 chloroform–methanol mixture and stored at −21 °C. Lipids were separated using two-dimensional (polar lipids) or one-dimensional (neutral lipids) thin-layer chromatography (TLC) [64,65] and quantified using standard compounds by the densitometry method (DENS software, version 5.1.0.2, Lenkhrom, St. Petersburg, Russia). To study the composition of fatty acids, the polar lipid fraction was isolated using one-dimensional TLC. The polar lipid spots at the start were scraped out and eluted with a mixture of chloroform:methanol (1:1); then the extract was evaporated and methanolysis was carried out using 2.5% H_2_SO_4_ in methanol for 2 h at 70 °C. The obtained methyl esters were analyzed by gas–liquid chromatography (GLC) on a Kristall 5000.1 gas–liquid chromatograph (Chromatec, Yoshkar-Ola, Russia) with an Optima-240, 60 m × 0.25 µm × 0.25 mm capillary column (Macherey-Nagel GmbH&Co, Düren, Germany). The temperature program used was from 130 °C to 240 °C at a rate of 5–6 °C/min. Identification was carried out using the Supelco 37 Component FAME Mix mixture of individual fatty acid methyl esters (Supelco, Bellefonte, PA, USA). The DU of the phospholipids was calculated according to the following equation [66]:

$$DU=\frac{1.0\times \left(\%\;monoene\;FA\right)}{100}+\frac{2.0\times \left(\%\;diene\;FA\right)}{100}+\frac{3.0\times \left(\%\;triene\;FA\right)}{100}+\frac{4.0\times \left(\%\;tetraene\;FA\right)}{100}$$

To determine the soluble carbohydrate composition of the mycelium, sugars were extracted with boiling water for 20 min four times. Proteins were removed from the resulting total extract [67]. The carbohydrate extract was further purified from charged compounds using a combined column with the Dowex-1 (acetate form) and Dowex 50 W (H^+^) ion exchange resins (Merck, Darmstadt, Germany). Carbohydrates and polyols composition was determined by GLC using trimethylsilyl derivatives obtained from the lyophilized extract [68]. The internal standard was α-methyl-D-mannoside (Merck, Darmstadt, Germany). Chromatography was carried out on a Kristall 5000.1 gas chromatograph (Chromatec, Yoshkar-Ola, Russia) with a ZB-5 30 m, 0.32 mm, 0.25 μm capillary column (Phenomenex, Torrance, CA, USA). The temperature was increased from 130 °C to 270 °C at a rate of 5–6 °C/min. Glucose, mannitol, arabitol, inositol, glycerol, erythritol, and trehalose (Sigma, St. Louis, MO, USA) were used as standards.

Results are presented as % of the dry weight and/or % of the total.

### 4.3. Cell Wall Isolation

Fungal CW was isolated following the modified Ram et al. method [15]. Approximately 1.5 g of wet mycelium was ground using a mortar and pestle, with the degree of disruption monitored under a Jenaval microscope (Carl Zeiss, Jena, Germany). Then, 20 mL of 1 M NaCl was gradually added, and the mixture was heated at 100 °C for 5 min to inactivate enzymes. The mixture was centrifuged at 5000 rpm, and the residue was extracted three times with 1 M NaCl, followed by three extractions with 20 mL of distilled water to remove intracellular contents. The remaining residue was treated with ethanol, centrifuged at 5000 rpm, and subsequently freeze-dried. The purification of the CW was monitored under a microscope with a magnification of 250× and 400×.

### 4.4. Analysis of Chitin and Glucan

The amount of chitin in the fungal CW was determined by measuring glucosamine using the Boas method with para-dimethylaminobenzaldehyde (CDH, New Delhi, India) [69], following the hydrolysis of 10 mg of CW with 10 mL of 6 N HCl for 6 h at 100 °C.

The amount of neutral polysaccharides in the CW was determined by analyzing the sugar content using the phenol-sulfuric acid method [70]. A hydrolysate was prepared by treating 10 mg of CW with 10 mL of 3 N HCl for 3 h at 100 °C.

### 4.5. Statistical Analysis

The experiments were carried out in triplicate, *n* = 3. The post hoc Dunnett test was used for pairwise comparison between control and CR or CFW. On all graphs, mean values ± standard error of the mean (SEM) are plotted. Statistically significant difference (*p* ≤ 0.05) is indicated by (*).

## Data Availability

The original contributions presented in this study are included in the article. Further inquiries can be directed to the corresponding author.
